# Replisome mechanics: lagging strand events that influence speed and processivity

**DOI:** 10.1093/nar/gku257

**Published:** 2014-05-16

**Authors:** Roxana E. Georgescu, Nina Yao, Chiara Indiani, Olga Yurieva, Mike E. O'Donnell

**Affiliations:** 1 Howard Hughes Medical Institute, Rockefeller University, 1230 York Avenue, NY 10065, USA; 2 Manhattan College, 4513 Manhattan College Pkwy, Riverdale, NY 10471, USA

## Abstract

The antiparallel structure of DNA requires lagging strand synthesis to proceed in the opposite direction of the replication fork. This imposes unique events that occur only on the lagging strand, such as primase binding to DnaB helicase, RNA synthesis, and SS B antigen (SSB) displacement during Okazaki fragment extension. Single-molecule and ensemble techniques are combined to examine the effect of lagging strand events on the *Escherichia coli* replisome rate and processivity. We find that primase activity lowers replisome processivity but only when lagging strand extension is inoperative. rNTPs also lower replisome processivity. However, the negative effects of primase and rNTPs on processivity are overcome by the extra grip on DNA provided by the lagging strand polymerases. Visualization of single molecules reveals that SSB accumulates at forks and may wrap extensive amounts of single-strand DNA. Interestingly SSB has an inter-strand positive effect on the rate of the leading strand based in its interaction with the replicase χ-subunit. Further, the lagging strand polymerase is faster than leading strand synthesis, indicating that replisome rate is limited by the helicase. Overall, lagging strand events that impart negative effects on the replisome are counterbalanced by the positive effects of SSB and additional sliding clamps during Okazaki fragment extension.

## INTRODUCTION

Chromosome duplication is performed by a multiprotein replisome machine that simultaneously replicates both strands of the parental duplex (1–5). DNA polymerases only extend DNA in the 3′–5′ direction, and therefore the antiparallel geometry of duplex DNA requires the two strands to be synthesized in opposite directions (3). The leading strand polymerase extends DNA in the same direction as helicase unwinding, while the lagging strand is copied in the opposite direction and is synthesized as multiple Okazaki fragments. Each Okazaki fragment is initiated by an RNA primer synthesized by primase, and RNA primers are eventually replaced with DNA for ligation of Okazaki fragments into a continuous daughter duplex.

Lagging strand synthesis requires several unique actions which could slow replisome speed or affect its stability and processivity. In bacterial systems primase interacts with the helicase to synthesize an RNA primer, ensuring that primers are formed at the replication fork junction (5–10). Primer synthesis proceeds in the opposite direction to helicase unwinding and thus the interaction of primase with the helicase may pause the replisome during RNA extension. SS B antigen (SSB) is specific to the lagging strand single-strand (ss) DNA and protects it from nucleases (3). However, the tightly bound SSB must be displaced during Okazaki fragment extension, and this energy expenditure by the polymerase may decrease the rate of the replisome. The lagging strand DNA must also form replication loops during extension of Okazaki fragments (11). Formation of DNA loops during replication has gained much experimental support in studies of replisomes from various sources (12–15). Any of these lagging strand specific events: priming, SSB displacement, and formation of replication loops, could conceivably take a toll on the processivity and/or rate of the replication fork.

Studies in the T7 phage system indicate lagging strand synthesis exacts a cost on the rate of replisome progression because primer synthesis by primase acts as a molecular brake that slows the replisome (16). However, other studies in the T4 and T7 phage systems contradict these findings (17,18). Reports in the *Escherichia coli* system also contradict one another; one report indicates that the processivity of the replisome is lowered by lagging strand events while another report concludes that it is enhanced by the lagging strand (19,20).

The current report combines single-molecule techniques and ensemble assays to study individual steps in lagging strand synthesis and their effect on the rate and processivity of the *E. coli* replisome. The *E. coli* replisome contains a helicase (the DnaB homohexamer) tightly associated with the replicative polymerase, DNA polymerase III (Pol III) holoenzyme, along with DnaG primase that transiently interacts with DnaB helicase for each priming event (2–4,21,22). The *E. coli* Pol III holoenzyme contains three Pol III cores and a single clamp loader that loads β clamps onto DNA for each polymerase (23–26). The leading strand only requires one Pol III-β clamp, while the lagging strand has multiple primed sites and utilizes the remaining two Pol III-β, as demonstrated both *in vitro* and *in vivo* (23–26).

This report demonstrates that primase action indeed lowers the processivity of the replisome, but only when lagging strand synthesis is inoperative. Okazaki fragment extension masks the negative effect of primase and significantly enhances replisome processivity. The greater processivity of the coupled leading/lagging strand replisome is based in the extra grip provided by additional polymerase-β clamp complexes engaged on the lagging strand. Our previous study showed that rNTPs decrease replisome speed by competing with dNTPs at the polymerase active site (27). Here we examine the effect on replisome processivity of adenosine triphosphate (ATP), the most prevalent rNTP in cells, needed for helicase, primase and clamp loading. We find that use of intracellular concentrations of ATP decreases replisome processivity as well as the rate of synthesis. Interestingly SSB, which binds the lagging strand, enhances leading strand replication in a species-specific fashion. This effect is based in contact of SSB with the χ subunit of the clamp loader within Pol III holoenzyme. Interestingly, visualization of fluorescent SSB during replication suggests that many copies of SSB accumulate at the fork. These SSB complexes appear to harbor extensive amounts of ssDNA, and may correspond to electron microscopy (EM) studies in the T7 phage system by the Griffith and Richardson groups that reveal large ssDNA-gp2.5 protein (i.e. T7 SSB) ‘bobbins’ at the replication fork (28). Pol III holoenzyme is faster on SSB coated ssDNA compared to the rate of leading strand synthesis, consistent with *in vivo* observations, and with biochemical reports in phage systems that helicase activity restricts the overall rate of fork progression (18,24). In overview, the current report demonstrates a network of steps in leading/lagging strand replication, some negative and some positive, that, in sum, counterbalance one another and enhance the rate and processivity of the replisome.

## MATERIALS AND METHODS

### Materials

Pol III subunits (α, ϵ, θ, τ, δ, δ′, χ, ψ, β), primase, SSB and DnaB helicase were purified as described (29). Wt Pol III* (Pol III core_3_τ_3_δδ′χψ), containing three polymerases, and monoPol III* (Pol III core_1_τ_1_γ_2_δδ′χψ) were reconstituted and purified from unassembled subunits as described (25). Pol III* minus χ (Pol III core_3_τ_3_δδ′ψ) was reconstituted as the wt Pol III* except χ subunit was omitted. Oligonucleotides were synthesized by Integrated DNA Technologies and gel purified. Glucose oxidase and catalase were from Sigma. Yo-Pro1 was from Invitrogen (Molecular Probes). Photo clear silicone-based elastomer (Sylgard 184) was from Dow-Corning (MI, USA). Lipids were from Avanti Polar Lipids, Inc.

### Single-molecule total internal reflectance fluorescence (TIRF) microscopy

TIRF microscopy was performed essentially as described (20). Flow cells were prepared using a photo-clear elastomer poured over a negative lithography mould to form a 2.5 mm X 50 μm channel. After the elastomer hardened, a rectangular block surrounding the channel was removed with a scalpel, and input and output ports were made at either end of the channel using a hole puncher. A plasma oven was used to weld the elastomer block to a coverslip into which diffusion barrier scratches had been etched using a diamond-tipped scribe. A lipid bilayer was formed on the glass surface inside the flow cell using a mixture of liposomes (DOPC, 8% DOPE-mPEG550 and 0.5% DOPE-biotin) as described (30). TIRF microscopy utilized an Olympus IX70 inverted microscope with a 60X TIRF objective (numerical aperature = 1.45), a motorized stage and motorized shutter. The motorized shutter permitted a 100 ms exposure every 1s. Buffer flow was adjusted to 100 μl/min using a syringe pump. Yo-Pro1 was excited using a solid state 488 nm laser at 1.5 mW and fluorescence emission was captured by a 512 × 512 pixel thin-backed EM CCD camera (Hamamatsu). Image collection and data work-up were facilitated using the Slidebook Software suite (Intelligent Imaging, Inc.).

The DNA substrate is a 100mer synthetic rolling circle containing a 5′ biotinylated 40 dT tail, prepared as described (29). Methods of replisome assembly, immobilization in the flow cell and visualization of leading strand replisome DNA products, were performed essentially as in (20). Briefly, we describe leading strand replisome reactions and then coupled leading/lagging strand replisome reactions. For leading strand replisomes, DnaB helicase (18.2 pmol, 365 nM) was assembled onto the rolling circle DNA (655 fmol, 13.1 nM) in 25 μl Buffer A (20 mM Tris-HCl, pH 7.5, 1 mM DTT, 40 μg/ml BSA, 4% glycerol) supplemented with 10 mM MgOAc_2_, 50 mM potassium glutamate and 100 μM ATP, followed by incubation for 30 s at 37°C. To this was added a 50 μl reaction containing Pol III* (675 fmol, 27 nM), β_2_ (1.85 pmol, 74 nM as dimer), 60 μM each dCTP and dGTP and 10 mM Mg(OAc)_2_ in Buffer A. After 1 min at 37°C, 0.5 μl of the reaction was added to 1 ml of Buffer B (8 mM MgOAc_2_, 60 μM each of dCTP and dGTP, and 50 nM Yo-Pro1 in Buffer A). The reaction was passed through the flow cell at 500 μl/min for 30 s, then at 10 μl/min for 30 s. DNA replication was initiated upon flowing (100 μl/min) Buffer A containing 60 μM of each dNTP, 0.5 mM ATP, 50 nM Yo-Pro1, 0.8% glucose, 0.01% β-mercaptoethanol, 0.57 U glucose oxidase, 2.1 U catalase and 100 nM each of five DNA 20mers that hybridize to the 100mer sequence of the leading strand product. Additional components, when present, were 250 μM each of CTP, GTP and UTP, 300 nM primase and additional ATP as indicated. Reactions were allowed to proceed for 20 min at 23°C, after which all DNA synthesis had stopped as described (20). Only DNA strands that were clearly separate from any others were used for analysis. The values reflect processivity (expressed in kb) obtained from a single-exponential fit ± SEM of the total number (*N*) of molecules analyzed. Experiments to determine the force of the flow used biotinylated λ DNA immobilized in the manner described above; a flow rate of 100 μl/min exerts a force of 1.45 pN and stretches λ DNA to 88% of its full contour length (20). All DNA lengths were corrected using this value.

Coupled leading/lagging strand replisome reactions were performed as described above except the five DNA 20mers are not present in the buffer flow, and the buffer contained 500 nM SSB_4_, 50 nM β_2_ and 300 nM primase. In some experiments, one DNA 20mer (100 nM) was substituted for primase in the buffer flow. In the presence of SSB, oligonucleotide annealing efficiency is reduced, and under the conditions used here, one primer anneals approximately every 1 kb (29).

To experimentally address whether any polymerase that dissociates, rebinds to another DNA from which the replication proteins had already dissociated, we performed the following control. We analyzed processivity histograms of DNA products localized at the input edge of the flow cell and compared them to processivity histograms of DNA products at the output edge of the flow cell. If Pol III dissociates from one DNA and re-associates with another DNA within a flow cell, it should do so progressively down the length of the flow cell, resulting in a processivity gradient along the length of the flow cell. However, our data analysis showed similar processivity histograms at both the input and output edges of the flow cell, indicating that re-association of dissociated replication proteins does not have a measurable impact on the observed results throughout the flow cell (Supplementary Figure S1).

### Ensemble replication reactions

Reactions contained 13 nM 100mer rolling circle substrate and 365 nM DnaB that were preincubated for 30 s at 37°C in 20 mM Tris-HCl, pH 7.5, 5 mM DTT, 40 μg/ml bovine serum albumin (BSA), 4% glycerol, 8 mM MgOAc_2_, 50 mM K-glutamate and 0.25 mM ATP. Then 27 nM Pol III* (TriPol unless indicated otherwise), 74 nM β_2_ and 60 μM each dCTP and dGTP were added, followed by a further 2 min incubation at 37°C and then the temperature was lowered to 25°C for 1 min. Replication was initiated upon adding 462 nM SSB_4_, 300 nM primase (unless indicated otherwise), 60 μM dATP, 20 μM ^32^P-dTTP, 750 μM ATP and 250 μM each of CTP, GTP and UTP (unless indicated otherwise). Timed aliquots were removed and quenched upon addition to an equal volume of 1% sodium dodecyl sulphate and 40 mM EDTA. Quenched reactions were divided; one half was analyzed for pmol ^32^P-nucleotide incorporated by spotting on DE81 filters followed by washing off unincorporated nucleotide and quantitation by liquid scintillation. The other half was analyzed in a 0.5% alkaline agarose gel followed by autoradiography using a Typhoon phosphoimager. Exceptions to these conditions are noted in the figure legends. To study the leading strand replisome, reactions were performed as described for the coupled replisome except primase was omitted. Some reactions utilize a DNA 20mer (53.2 nM) in place of primase, as described (29). Reactions on primed SSB coated ssDNA were performed using 5.4 kb circular ϕX ssDNA (1.5 nM) to which was annealed a 5′ ^32^P end-labeled DNA 30mer, and otherwise the reactions were as described above for rolling circle leading/lagging strand replication. Quenched reactions were divided; one half was analyzed for pmol ^32^P-nucleotide incorporated using DE81 filters, and the other half was analyzed in a 0.8% alkaline agarose gel followed by autoradiography using a Typhoon phosphoimager.

## RESULTS

### Primase activity with rNTPs decrease replisome processivity in the absence of lagging strand synthesis

**Figure 1.**
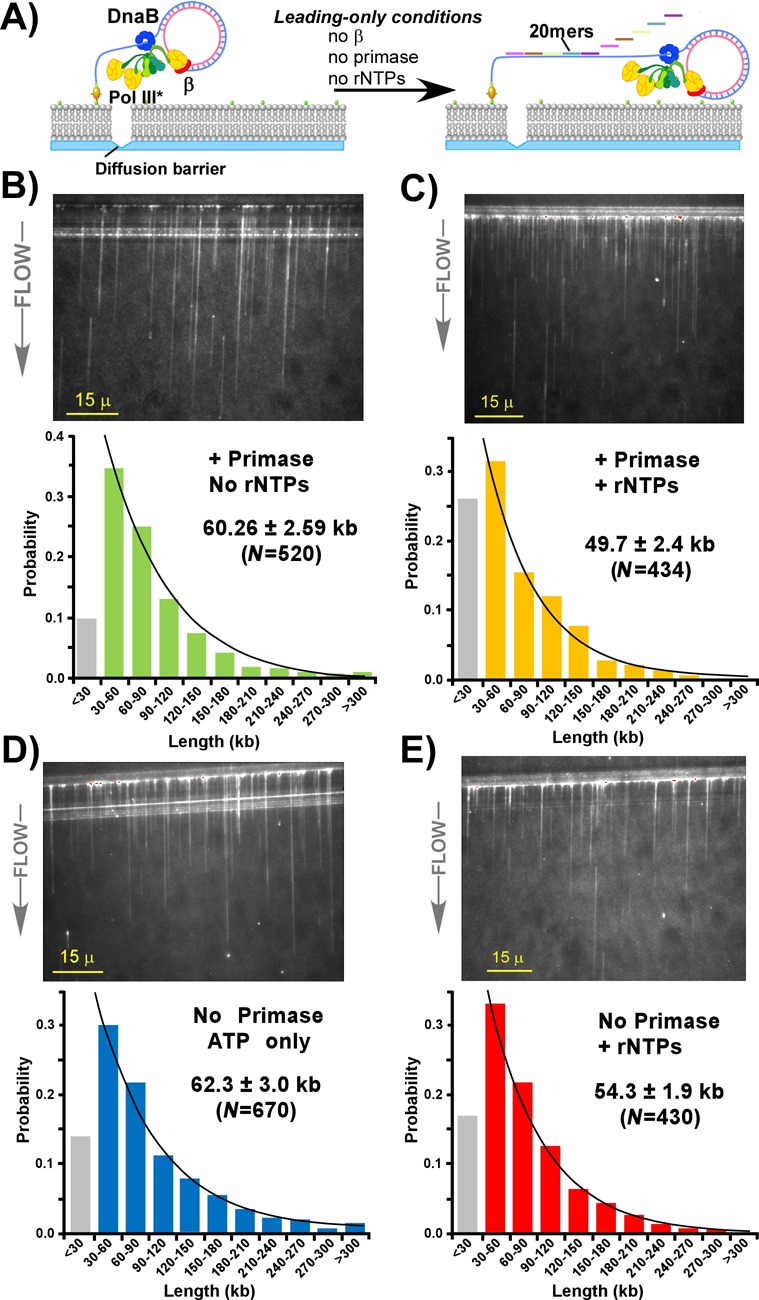
Single-molecule TIRF microscopy of leading strand replisome products. Panel **A**: scheme of the assay to monitor synthesis by a leading strand replisome. Left: DnaB helicase (blue), Pol III* (composed of three Pol III cores (yellow) attached to a central clamp loader (green)) and the β clamp (red) are assembled onto a 5′ biotinylated rolling circle substrate. Only the leading strand polymerase is attached to a β clamp. The replisome/DNA complex is then attached to a lipid bilayer in a flow cell using neutravidin to couple the DNA to biotinylated lipids. The interruption in the bilayer represents a diffusion barrier along which the replisome/DNA complexes align in a hydrodynamic flow. Right: replication is initiated upon flow of a buffer containing ATP and lacking additional β. Under these conditions only the leading strand is synthesized. The buffer flow contains a mixture of five DNA 20mers that anneal end-to-end and convert the ssDNA to duplex DNA which is visualized by an intercalating dye (YoPro1) present in the buffer flow. Panels **B** and **C** show histograms of DNA length of products observed using primase with or without three rNTPs (rGTP, rCTP, rUTP), respectively. An example visual field is shown above each histogram, but many fields of view must be examined in order to identify sufficient numbers (*N*) of individual DNA molecules to create each histogram. Panels **D** and E are histograms of DNA lengths observed in the absence of primase, with or without three rNTPs. Numbers represent the single-exponential fit ± SEM of the total number (*N*) of molecules analyzed.

The *E. coli* replisome is highly processive and quickly forms DNA products that are too long to resolve in an agarose gel (31). Therefore we measured the processivity of the replisome using single-molecule TIRF microscopy that directly visualizes long DNA products in a microscope (20). The DNA substrate for these studies is a rolling circle template attached to a supported lipid bilayer formed in a customized flow cell. The 5′ tail of the rolling circle contains a biotin moiety enabling it to be immobilized on the lipid bilayer through a neutravidin bridge to biotinylated lipids (see illustration in Figure 1A). The force of the hydrodynamic flow pushes the DNA-lipid complex to a diffusion barrier etched in the glass surface, concentrating numerous DNA molecules at a defined position within the flow cell and enables many molecules to be examined simultaneously in one field of view. Replication in a flow cell will stop when an essential replisome component dissociates, because it will be quickly washed away in the buffer flow. The final length of the DNA products, when averaged, is a measure of replisome processivity.

To reconstitute the replisome on DNA, DnaB helicase is first assembled onto the DNA, then Pol III* ((Pol III core)_3_τ_3_δδ′χψ) and the β clamp are added, but dTTP and dATP are absent to prevent elongation. The replisome-DNA complex is then passed into the flow cell for attachment to the lipid bilayer. After attachment, replication is initiated upon flowing a buffer that contains the four dNTPs, four rNTPs and a fluorescent intercalating dye to visualize the DNA products, along with primase, SSB and β that are needed in continuous supply during replisome action (20). To examine the effect of primase–DnaB interaction and primase activity on replisome processivity, the experiments of Figure 1 study a replisome that only synthesizes the leading strand (i.e. a ‘leading strand replisome’). Lagging strand synthesis is prevented by omitting β from the buffer flow. The leading strand replisome synthesizes a long ssDNA ‘tail’ as it proceeds numerous times around the 100mer rolling circle DNA. Since the ssDNA product does not bind the fluorescent intercalator, we include a mixture of five DNA 20mers that anneal end-to-end over the repeated 100mer sequence to convert the ssDNA product to dsDNA and thus enable the DNA product to be visualized (see illustration in Figure 1A).

To measure processivity, replication is allowed to proceed for 20 min, sufficient time for all replisomes to dissociate and synthesis to stop (20). The experiments of Figure 1, Panels B/C, measure the processivity of the leading strand replisome in the presence of primase with all four rNTPs or with only ATP in the buffer flow (i.e. 0.5 mM ATP is present to fuel DnaB helicase). Under these conditions, primase should still bind to DnaB, but cannot synthesize RNA primers. Examples of visual fields are shown in Figure 1B and C along with the histogram quantitation of individual products. Histograms are constructed from the analysis of numerous visual fields, because only those DNA molecules that start from the diffusion barrier and are separated from other strands are measured. The results show that conditions of active priming (+ primase, + rNTPs) lower replisome processivity from 60.3 kb (primase, ATP only, Panel B) to 49.7 kb (primase + rNTPs, Panel C). To determine the effect of rNTPs on processivity in the absence of primase, experiments were performed without primase and either without rNTPs (62.3 kb) or with ATP only (54.3 kb) (Figure 1D and E, respectively). The results indicate that rNTPs lower replisome processivity (54.3 kb), but the effects of rNTPs in the absence of primase are not firm because the observed processivity values are within the extremes of the experimental error. Comparison of Panels B and D indicate that when primase is inactive (only ATP is present), it has no significant effect on replisome processivity under conditions that do not permit lagging strand synthesis (no β).

### Okazaki fragment extension enhances replisome processivity

**Figure 2.**
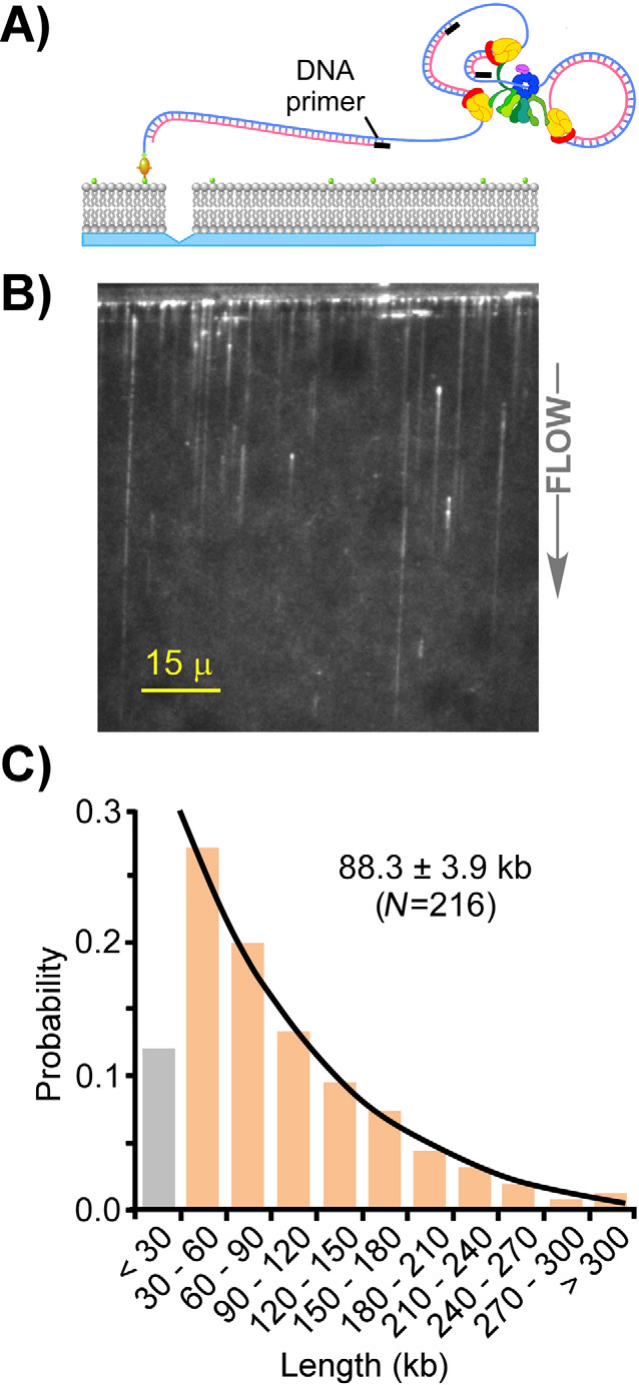
Coupled leading/lagging strand products using DNA oligos in place of primase. Panel **A**: illustration of the coupled leading/lagging strand replisome. Either primase (makes RNA primers) or DNA primers (shown) initiate Okazaki fragment synthesis for coupled replication of both strands of DNA. DNA primers are used in place of primase for Okazaki fragment synthesis. Panel **B**: visual field of products resulting from a coupled leading/lagging strand replisome using DNA primers to prime Okazaki fragment synthesis. Autofluorescence from the diffusion barrier is visible at the top. Primase and rNTPs were omitted from the reaction, and the β clamp (50 nM) was present in the buffer flow. Panel **C**: DNA length distribution histogram of a coupled leading/lagging strand replisome using DNA primers in place of primase and rNTPs for lagging strand replication.

Next we examined the processivity of the coupled leading/lagging strand replisome. Our previous studies on this subject showed that the coupled replisome, with rNTPs and primase present, has a processivity of 86.5 kb (27). Since primase and rNTPs lower processivity of the leading strand replisome (e.g. Figure 1), we wished to examine the effect of Okazaki fragment extension on coupled leading/lagging strand replisomes in the absence of primase and rNTPs. Primase and three rNTPs (rG, rC, rU) can be circumvented upon adding DNA 20mer oligonucleotides to prime the lagging strand (29). In the experiment of Figure 2, we omitted primase and rG, rC and rU (three rNTPs), and used a DNA 20mer oligonucleotide to enable coupled synthesis in the absence of primase (illustrated in Figure 2A). Under the conditions used here, the oligonucleotide anneals approximately once every 1 kb, producing Okazaki fragments of about 1 kb (29). The results of Figure 2B show an example ‘DNA curtain’ produced by the coupled leading/lagging replisome. Control experiments confirm that the leading strand is synthesized and is available for hybridization for the 20mer oligos (Supplementary Figure S2). The histogram analysis of DNA curtains (Figure 2C) yields a processivity value of 88.3 kb, significantly greater than the 49.7 kb observed for the leading strand replisome in the presence of priming activity, and also greater than the 62.3 kb processivity of the leading strand replisome in the absence of primase and three rNTPs. Hence, the negative effects of primase and rNTPs on replisome processivity are masked by the extra grip to DNA conferred by the lagging strand clamp-polymerase complexes.

### DNA looping does not decrease the rate of fork progression

We next examined the rate of the replisome in ensemble assays. For rate studies, use of a 100mer synthetic rolling circle provides two advantages over large 7.2 kb M13 rolling circle substrates. First, the 100mer has no dA residues on the inner circle (leading template strand) enabling the leading strand product to be specifically labeled using ^32^P-dTTP. Second, extension products of the 100mer DNA start from 100 bp and can be accurately measured to 12 kb, while extension of a 7.2 kb rolling circle DNA can only be accurately measured for an additional 5 kb. *In vivo* analysis and *in vitro* rolling circle studies of the *E. coli* replisome show it is exceedingly rapid (>600 bp/s at 37°C) (32,33). To increase the accuracy of the rate measurements we slowed replisome movement about 2-fold by lowering the temperature to 25°C, similar to the temperature used in the single-molecule experiments.

**Figure 3.**
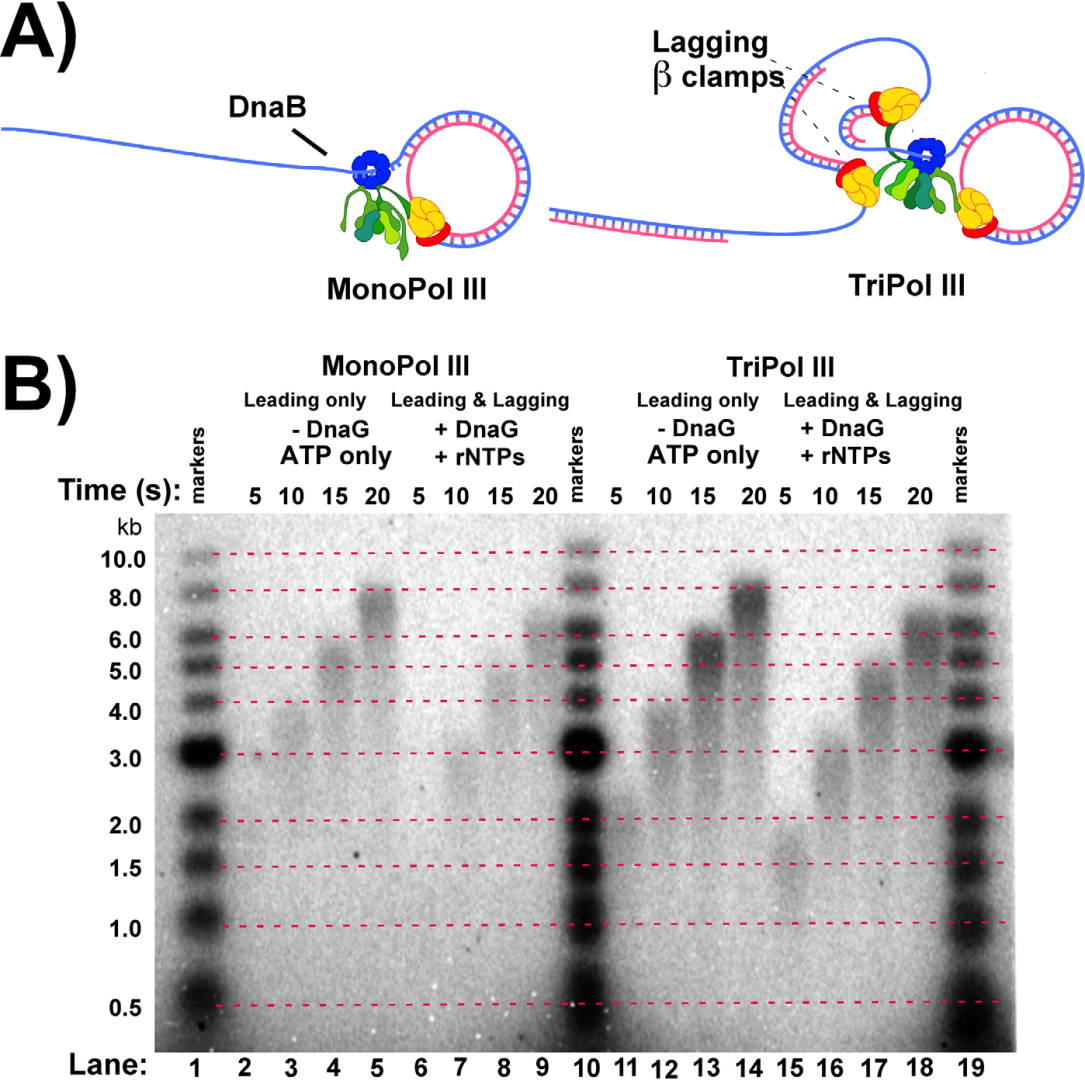
Comparison of MonoPol III and TriPol III replisomes. (**A**) Replisomes were assembled on the rolling circle substrate using either MonoPol III* or TriPol III*, along with DnaB and β. (**B**) Replication was performed using either MonoPol III or TriPol III replisomes in the presence or absence of primase and/or three rNTPs (rG, rC and rU), as indicated above the autoradiogram.

In the experiment of Figure 3, we compare rates of replication using either MonoPol III* ((Pol III core)_1_ τ_1_γ_2_δ_1_δ′_1_χ_1_ψ_1_) or TriPol III* ((Pol III core)_3_τ_3_δ_1_δ′_1_χ_1_ψ_1_) (Figure 3A). These polymerase stoichiometries are based in the fact that the clamp loader contains three products of the *dnaX* gene. This gene produces a mixture of full length τ subunit and the shorter γ subunit which is truncated by a translational frameshift. Pol III* can be reconstituted such that it contains only one τ, two γ, and thus one Pol III core (MonoPol III*) or three τ, no γ, and thus three Pol III core (TriPpol III*) (25). The TriPol III* is the wt form, also referred to as Pol III holoenzyme. Replication forks constituted using the MonoPol III* can only form leading strands at a replication fork, although excess unbound MonoPol III* can fill in lagging strand fragments independent of the replisome. Hence, replication using MonoPol III* will not form DNA loops during replisome progression, while replisomes containing the TriPol III* contain two additional polymerases which have been shown to function on the lagging strand and thus form Okazaki fragment loops (15,20). Comparison of rates by MonoPol III and TriPol III replisomes will reveal whether formation of DNA loops decrease the rate of the coupled replisome. The results demonstrate that the two replisomes travel at similar speeds, and that the presence of primase/rNTPs slows the rates of each replisome to the same extent (Figure 3B). Hence, the rate of the replisome is decreased by primase and/or rNTPs, and the source of the rate decrease is not associated with DNA looping during lagging strand replication.

### Lagging strand synthesis ‘*per se*’ does not decrease replisome rate

**Figure 4.**
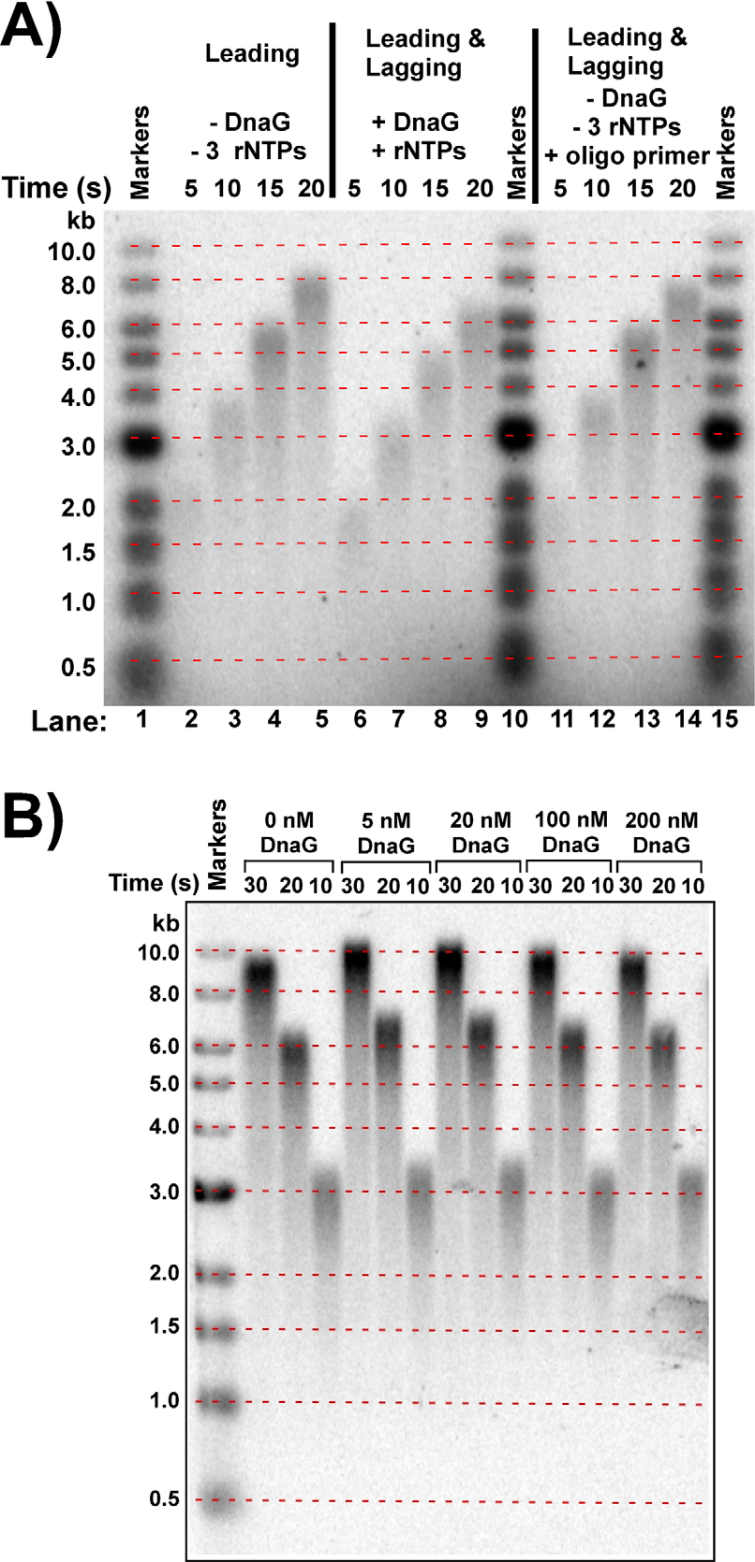
Lagging strand synthesis ‘*per se*’ does not slow the rate of fork progression. (**A**) Ensemble assays that compare the rate of the leading strand replisome with rates of coupled leading/lagging strand replisomes. Lanes 1, 10 and 15 are size standards, as indicated to the left of the gel. The horizontal red dashed lines are drawn between the same size markers on either side of the gel as an aid to identify DNA length differences among the DNA products. Lanes 2–5 is a leading strand replisome (no primase and no CTP, GTP and UTP, but ATP is present for DnaB). Lanes 6–9 is a coupled leading/lagging replisome using primase and rNTPs to prime the lagging strand. Lanes 11–14 are coupled leading/lagging replisome reactions using DNA primers to prime the lagging strand (no primase and no CTP, GTP, UTP). (**B**) Coupled leading and lagging strand reactions in the presence of four rNTPs and the indicated amounts of primase.

To determine if lagging strand synthesis inhibits the replisome in the absence of primase and with ATP only (no rG, rC, rU), we used the 20mer primer to initiate Okazaki fragments and compared this reaction to the rate of the leading strand replisome. The 20mer primer was used under conditions that enable lagging strand synthesis in the presence of SSB as described in our earlier study (25). The result, in Figure 4A, shows that the rate of the coupled leading/lagging strand replisome using DNA primers (lanes 11–14, 336 ntd/s) is similar to the rate of the leading strand replisome in the absence of lagging strand synthesis (lanes 2–5, 363 ntds/s). Hence, lagging strand synthesis *per se* does not inhibit the rate of the replisome. The control, in lanes 6–9 of Figure 4A, confirms that the presence of primase and four rNTPs decreases the rate of the replisome (286 ntds/s). Next, we examined whether the concentration of primase inhibits the rate of the coupled leading/lagging strand replisome. Studies by the Marians group have demonstrated that increasing primase results in more frequent priming and thus shorter Okazaki fragments (33). In the experiment of Figure 4B, primase was titrated into replisome-mediated rolling circle reactions in the presence of rNTPs. Titration of primase into reactions containing rNTPs shows very little effect of primase and RNA primer synthesis on the slower replisome, implying that slower forks are due to the rNTPs themselves. We have shown previously that rNTPs decrease the rate of the replisome by competing with dNTPs at the polymerase active site (27). Considering that the experiments of Figures 3 and 4 rule out DNA looping and primase as inhibitors of replisome rate, it would appear that the inhibition by rNTPs is the sole reason for a decrease in the rate of the coupled *E. coli* replisome. Next we examined the effect of rNTPs on DnaB rate and the processivity of the replisome.

### Effect of rNTPs on the replisome

**Figure 5.**
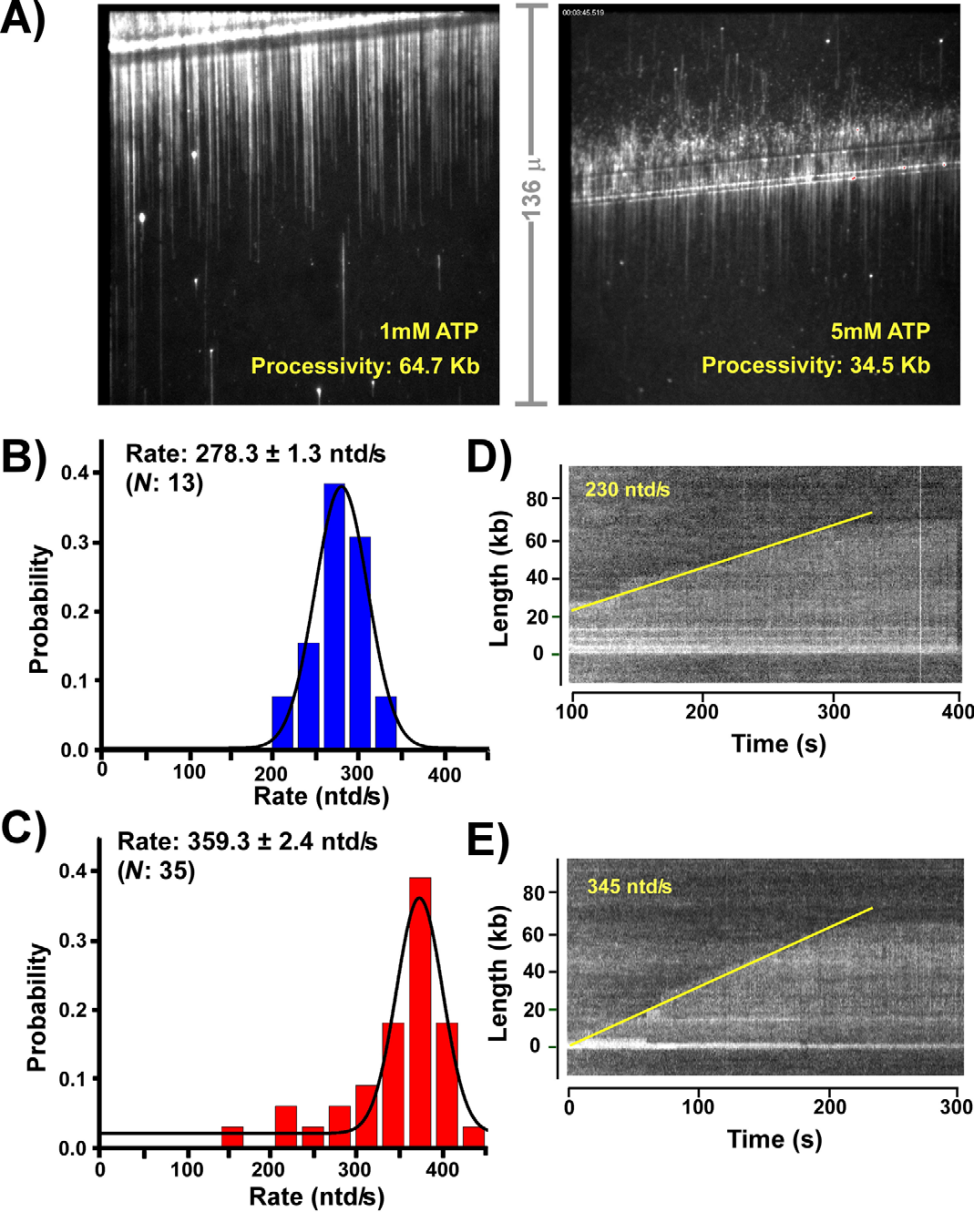
The effect of ATP on the rate and processivity of replication forks. Panel **A**: representative fields of view of single-molecule replication reactions performed on the coupled leading/lagging strand replisome in the presence of either 1 mM ATP (left) or 5 mM ATP (right). Details are in Experimental Procedures. Panels **B**–**E**: single-molecule studies follow the rate of individual coupled replisomes in real-time in the presence of either 1 mM ATP or 5 mM ATP. Panel B shows a distribution histogram of the rate of growth of DNA products using 5 mM ATP. Panel C is similar to Panel B, except using 1 mM ATP. The rates given in Panels B and C represent the single-Gaussian fit ± SEM of the total number (*N*) of molecules analyzed. Panels D and E are representative kymographs of DNA product synthesized by an individual replisome in the presence of either 5 mM ATP (Panel D) or 1 mM ATP (Panel E).

rNTPs are required substrates for the helicase as well as the primase and clamp loader. In the experiment of Supplementary Figure S3, we examine the effect of each rNTP on replisome rate. While DnaB can use each rNTP, it is possible that one rNTP supports faster replisome progression than other rNTPs. In this case, a mixture of four rNTPs might result in competition among rNTPs and a lower than optimum DnaB rate. However, the result shows that each of the four rNTPs is equally capable of supporting DnaB unwinding, and thus rNTPs do not act on the helicase to slow the fork (Supplementary Figure S3). In the experiment of Figure 5, we use single-molecule TIRF microscopy to examine the effect of rNTPs on replisome processivity, as well as the rate. We chose to focus on ATP, since ATP is the most abundant rNTP in *E. coli* (3.6 mM), presumably because it is used as an energy source in addition to its role as a nucleic acid precursor (34). In Figure 5A, we test the effect of 1 mM and 5 mM ATP on the processivity of the replisome. The results show that processivity diminishes as ATP is increased from 1 mM to 5 mM, as can be directly observed from comparison of the DNA curtains in Figure 5A. The histogram distribution analysis of DNA product length gives processivities of 64.7 kb and 34.5 kb for 1 mM and 5 mM ATP, respectively (Supplementary Figure S4). Primase is not present in the assays of Figure 5A and therefore the observed decrease in replisome progression is not due to primer synthesis.

Single-molecule real-time observations were used to measure the rate of the replisome at 5 mM and 1 mM ATP (Figure 5B and C, respectively). At 1 mM ATP, the observed rate is 359.3 ± 2.4 ntd/s, and increasing ATP to 3 mM results in a lower rate, 278.3 ± 1.3 ntd/s. Example kymographs of individual replisomes are shown in Figure 5D and E for 3 mM and 1 mM ATP, respectively. Collectively, the decrease in both rate and processivity with increasing ATP suggests that lower processivity may be explained, at least in part, as a consequence of slower replisome progression, in which the dissociation rate (*k*_off_) of an essential replisome component (i.e. Pol III, β, or DnaB) occurs with the same kinetics irregardless of the ATP concentration. Hence, given the slower rate of a replisome at elevated ATP, less DNA is synthesized in the time it takes for a replisome protein to dissociate compared to the length of DNA synthesized at lower ATP.

### SSB stimulates the replisome in a species-specific fashion

**Figure 6.**
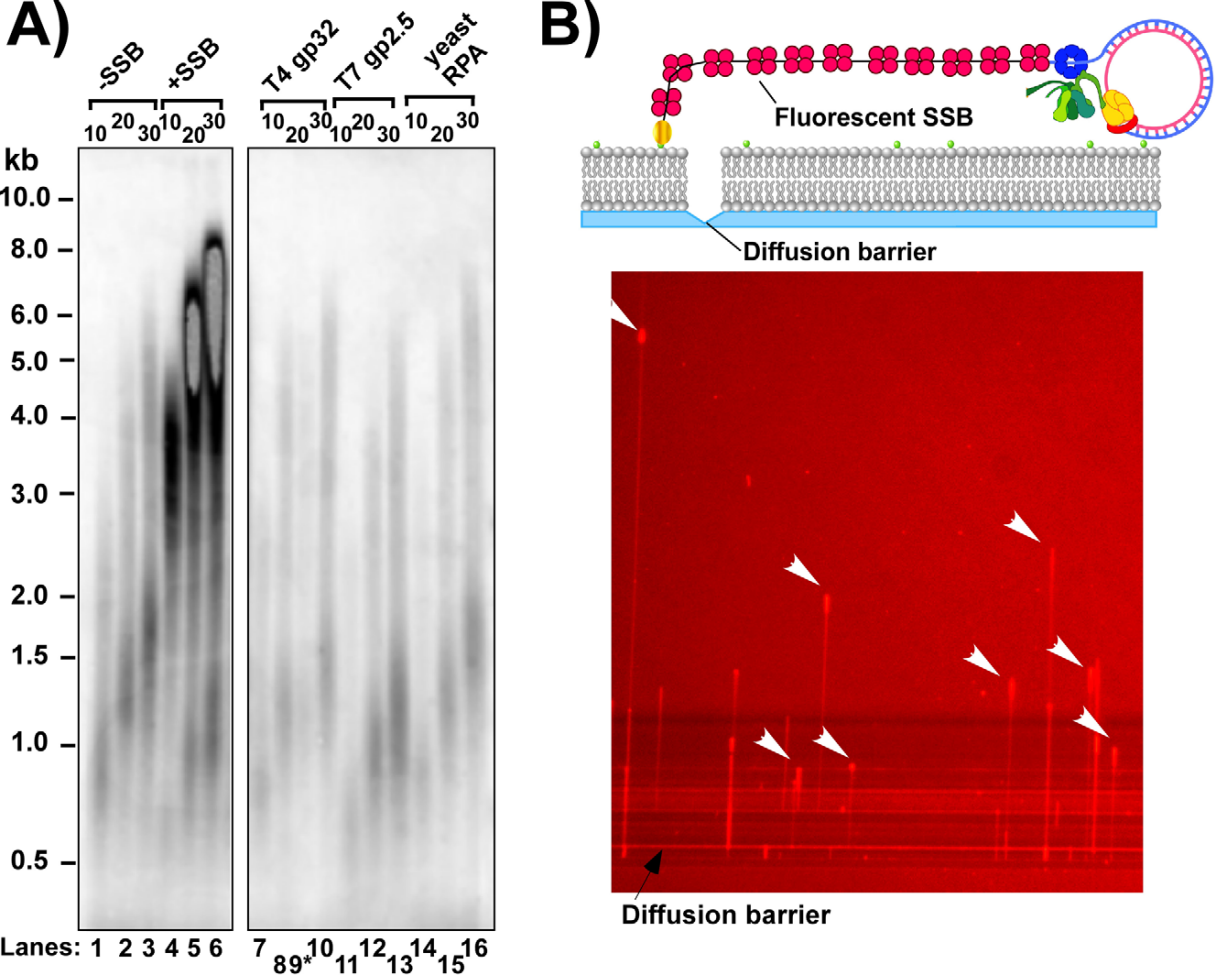
Effect of SSB on the leading strand and visualization of SSB-ssDNA ‘bobbins’ at the fork. Panel **A**: replication was performed on the 100mer rolling circle substrate using the TriPol III replisome which performs coupled leading/lagging replication, and either: no SSB (lanes 1–3), *E. coli* SSB (lanes 4–6), T4 gene 32 protein (lanes 7–10; lane 9* is a spill over from lane 8), T7 gene 2.5 protein (lanes 11–13) and yeast RPA (lanes (14–16). Panel **B**: single-molecule microscopy of the MonoPol III replisome (as in Figure 3) in the presence of Texas red-labeled *E. coli* SSB. The MonoPol III replisome performs only leading strand replication. The diffuse red background is due to the Texas red-SSB in the buffer flow (i.e. epifluorescence is used). Replication forks are located at the tips of the growing strands (arrows) which appear more intense then the filament and may contain many copies of Texas red-SSB.

The experiment of Figure 6A uses the TriPol III* to enable coupled leading/lagging replication and shows that leading strand synthesis is markedly stimulated by SSB (Figure 6A, compare lanes 1–3 with 4–6). SSB is thought to function only on the lagging strand, as the direct connection of the leading strand polymerase to DnaB is presumed to leave too little ssDNA for SSB to bind the leading strand (31). To stimulate the leading strand, SSB bound to the lagging strand must either stimulate DnaB and/or the leading strand polymerase. In Figure 6 we investigate whether SSB stimulation of the replisome is specific to *E. coli* SSB, or whether heterologous SSBs can substitute. We used three different heterologous SSBs (e.g. T4 gp32, T7 gp2.5 and yeast RPA) and none of them stimulated the replisome. The fact that heterologous SSBs are not as efficient as *E. coli* SSB suggests that SSB stimulation is mediated by specific protein–protein interaction. The χ subunit of Pol III holoenzyme is known to bind SSB (35,36). To determine whether χ-to-SSB contact underlies the SSB stimulation, we compared replisomes using either wt Pol III holoenzyme or Pol III holoenzyme lacking the χ subunit (Supplementary Figure S5). The results show that the replisome with wt Pol III holoenzyme (containing χ) is stimulated by SSB to a much greater extent than replisomes using Pol III holoenzyme lacking χ. Hence, χ–SSB contact may underlie stimulation of the leading polymerase. This conclusion is supported by an earlier observation of Pol III action in strand displacement synthesis, in which Pol III-β (without DnaB) is stimulated by χ binding to SSB bound to the displaced strand (37).

### SSB bobbins at a moving replication fork

EM studies reveal that the T4 SSB gene 32 protein forms a higher-order (oligomeric) structure on the lagging strand that the authors refer to as a ‘bobbin'. They propose that the ssDNA of the lagging strand is coiled around multiple gene 32 proteins, as a way of organizing the ssDNA. To determine if *E. coli* SSB also forms higher-order structures on ssDNA during replication, we performed single-molecule experiments using SSB labeled with a Texas red fluorophore to visualize SSB on DNA. Rolling circle replication was performed in the absence of primase to enable binding of sufficient Texas red-SSB to visualize. To excite the Texas red-SSB we used a mercury lamp instead of the laser (i.e. epifluorescence). Real-time visualization shows long growing filaments of Texas red-SSB on lagging strand ssDNA (Figure 6B and Supplementary movie S1). The growing tips (i.e. replication forks) of most of the molecules contain a bright red spot, presumably an accumulated mass, or ‘bobbin', of Texas red-SSB-ssDNA at the replication fork. Real-time observations show that the red SSB filaments periodically undergo a burst of extension that is over 10 times faster than DNA synthesis, suggesting an accumulated store of ssDNA that can periodically and suddenly unravel (Supplementary Figure S6).

### Extension on a lagging strand model ssDNA is faster than the leading strand replisome

**Figure 7.**
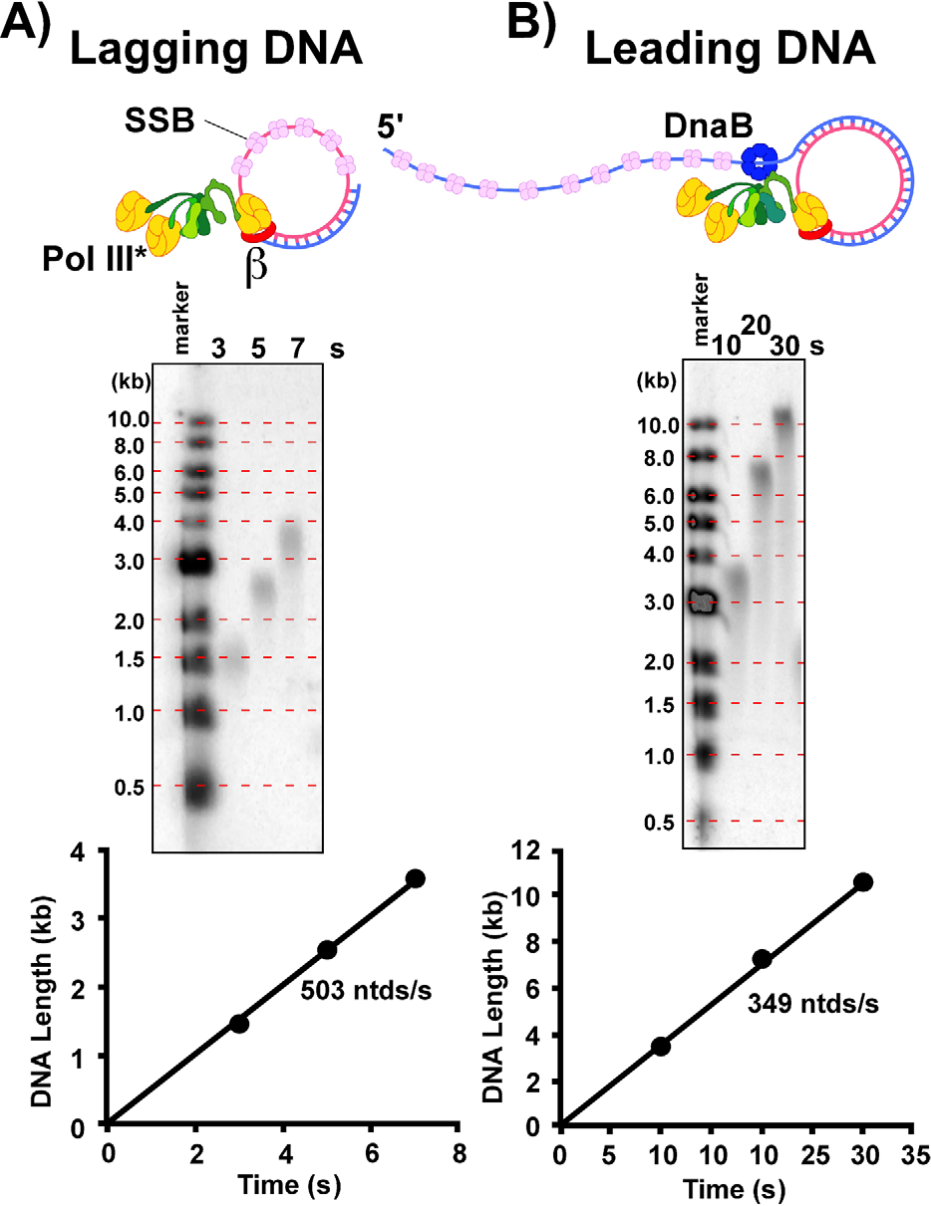
Synthesis on a lagging strand model DNA is faster than the leading strand. Panel **A**: the rate of Pol III holoenzyme on SSB coated ssDNA, a model of the lagging strand. Pol III*-β is assembled onto 5′ ^32^P-labeled primed ϕX ssDNA, then synchronous replication is initiated as described in Experimental Procedures. Timed aliquots are analyzed in an alkaline agarose gel. Panel **B**: rolling circle reactions as described in Experimental Procedures (the rolling circle substrate was 5′ labeled with ^32^P. The four rNTPs and dNTPs were present in both reactions. Reaction products are analyzed in alkaline agarose gels. Peak intensities from laser scans of each lane are plotted versus time to obtain rates of elongation.

SSB stimulates its cognate polymerase on ssDNA by melting secondary structures (38). However, displacement of tightly bound SSB requires energy and would probably slow the intrinsic speed of a polymerase on DNA lacking hairpins. The leading polymerase does not need SSB because it receives newly unwound ssDNA directly from the helicase (31). Hence, the leading strand polymerase does not need to displace SSB and could conceivably travel faster than the lagging strand polymerase, which must displace tightly bound SSB. Measurements of leading and lagging strand replication by the Patel group in the T7 system reveal that the rate of the helicase limits the leading strand polymerase, and that the lagging strand polymerase is faster than the leading strand (18). The same applies to the *E. coli* system, as demonstrated by the experiments of Figure 7. We compared the rate of the leading strand replisome (Figure 7B) with the rate of primer extension on an SSB coated 5.4 kb primed ϕX ssDNA (Figure 7A), a model of lagging strand synthesis. Both reactions include a preincubation step with limiting dNTPs to allow time for proteins to assemble on DNA, and then synchronous elongation is initiated upon adding the remaining dNTPs and the four rNTPs. The result shows that the rate of synthesis on SSB coated ssDNA (503 ntd**/**s) is greater than the leading strand replisome (350 ntd/s), indicating that the rate of helicase unwinding limits replisome speed. Addition of DnaB to primer extension assays after binding SSB to the ssDNA has no effect on the rate of synthesis.

## DISCUSSION

This report examines the effect of lagging strand specific actions on the rate and processivity of replisome progression. Events unique to the lagging strand include the interaction of primase with DnaB, RNA primer synthesis, DNA synthesis on SSB coated ssDNA and formation of a DNA loop for each Okazaki fragment. The findings of this report reveal steps in lagging strand synthesis that influence the rate and processivity of the replisome. Some steps inhibit replisome rate or processivity while others have positive consequences. Taken together, a picture emerges of a network of reactions that collectively stimulate both speed and processivity of the coupled replisome. For example, primase has a negative effect on processivity in the presence of rNTPs provided that lagging strand synthesis is not permitted, but primase does not appear to affect processivity in the absence of rNTPs whether the lagging strand is synthesized or not. The primase effect is masked during coupled synthesis, because the extra clamps on DNA required for lagging strand replication provide ample extra grip to overcome replisome instability caused by primase. In another example, SSB tightly binds lagging strand ssDNA and must be displaced during Okazaki fragment synthesis which conceptually may lower the intrinsic rate of a DNA polymerase. However, we find that SSB bound to the lagging strand stimulates leading strand synthesis and does so in a species-specific fashion. In addition, the Pol III holoenzyme is faster on SSB-coated ssDNA (e.g. the lagging strand) than the leading strand polymerase of a replisome, because DnaB helicase limits the rate of the replisome. Hence, any decrease in polymerase rate due to SSB displacement is without consequence at a moving replication fork. In addition, study of DNA looping by comparison of MonoPol III and TriPol III replisomes shows that DNA loops that form during Okazaki fragment extension do not affect the leading strand rate. In sum, both the speed and processivity of the *E. coli* replisome are unencumbered by lagging strand synthesis, and in fact are aided by it.

The current study demonstrates that elevated ATP concentration slows the replisome and also decreases the processivity (Figure 5). Further, comparison of reactions with and without rGTP, rCTP and rUTP demonstrates that rNTPs decrease replisome processivity in the absence of lagging strand replication (Figure 1). Each rNTP fuels the helicase rate to the same extent (Supplementary Figure S3), making helicase an unlikely target of rNTP mediated inhibition. These findings support the conclusion of an earlier study that the DNA polymerase is the target of rNTP mediated slowdown (27). An earlier single-molecule study observed that primase decreased replisome processivity in the absence of lagging strand synthesis, consistent with studies reported here (19). However, the earlier study did not observe that rNTPs affect the processivity or rate of the leading strand replisome, probably because different experimental conditions were used. The earlier study omitted SSB, primase and the χψ subunits of Pol III holoenzyme to help prevent lagging strand synthesis, and DnaC was included in the flow to facilitate helicase loading. The absence of SSB and χ probably explain the difference, as the current report shows that SSB is needed to attain the full rate of leading strand synthesis and localizes this effect to a χ-to-SSB interaction. In addition, DnaC, which facilitates DnaB loading, can have inhibitory effects on DnaB when DnaC is in the ATP bound state (39).

Despite the negative consequences of rNTPs on replisome progression, rNTPs may have a downstream positive effect on the overall fidelity of replication. Although the vast majority of polymerase-rNTP binding events do not lead to misincorporation of rNMP into DNA, the sheer number of these binding events leads to a low level of rNMP incorporation (27). Although *E. coli* has a methyl-directed mismatch repair system, rNTP incorporation is suggested to facilitate mismatch repair in eukaryotes and in most bacteria, by marking the newly replicated strand (27,40,41). Evidence suggests that the low level of rNMP incorporation may direct the specific nicking of the newly replicated strand by the RNaseH involved in repair of rNMPs in DNA (41).

Early studies in the T4 phage system by the Alberts group concluded that DNA loops are formed during lagging strand Okazaki fragment extension because the lagging strand polymerase remains attached to the moving replisome (42). DNA loops have since been directly observed by EM in both the phage T4 and phage T7 systems (13,14), and by the dynamic movement of beads attached to the lagging strand in single-molecule studies in the phage T7 system (12). The continual association of the lagging strand polymerase with the replisome is also supported by single-molecule studies of rolling circle replication performed in the absence of Pol III* in the buffer flow (e.g. Figure 2). Without Pol III* in the buffer flow the lagging Pol III must constantly remain with the replisome, forming a DNA loop for each Okazaki fragment because if the lagging Pol III were to dissociate it would be carried away in the buffer flow and production of dsDNA would abruptly stop.

This report makes two interesting observations on the roles of SSB in replication. First, SSB stimulates the replisome in a species-specific manner. SSB stimulation requires the χ subunit of the Pol III replicase, which is known to bind SSB (35,36). Hence the leading strand polymerase benefits from the SSB bound to the lagging strand (Figure 6A). This inter-strand communication is also observed in the absence of DnaB in which strand displacement is catalyzed by Pol III in a reaction that depends on contact of χ to SSB bound to the displaced strand (37). We note that SSB stimulation of replisome rate is likely amplified in reactions that utilize small rolling circle DNAs compared to larger 7.2 kb M13 rolling circles (31). Perhaps small circular DNAs have torsional constraints that larger DNAs lack. Hence the use of a small DNA rolling circle more clearly unveils the effect of SSB on replisome rate.

We also directly visualize accumulations of SSB-ssDNA at the tip of a moving replication fork (Figure 6B and Supplementary movie 1). A large amount of ssDNA must be packaged within these higher-order structures because when they spontaneously unravel they undergo a burst of extensive and very rapid expansion. These structures probably resemble the SSB-ssDNA ‘bobbins’ previously observed by EM studies in the T4 system (28). The authors suggested that the bobbins of T4 gene 32 protein function is to wrap and organize the lagging strand ssDNA as it is produced by helicase unwinding, compacting it and protecting it from nucleases.

The several unique features of lagging strand synthesis (i.e. primase, DNA looping and SSB displacement) stand in sharp contrast to the simplicity of leading strand synthesis in which the polymerase simply follows the helicase as it unwinds parental DNA. The unique actions required for lagging strand synthesis call into question how the lagging strand keeps pace with the leading strand. The simplest explanation is that the rate of helicase unwinding limits the rate of the leading strand. In fact, it has been noted that T4 replicative polymerase is inefficient in strand displacement and requires a helicase, an arrangement in which the rate of leading strand synthesis is dictated by the rate of the helicase (43). Helicase limited replisome progression also has experimental support in the T7 system (16,18), and has been proposed for the *E. coli* system (19). As predicted by these previous studies, the current report measures the rates of the *E. coli* Pol III holoenzyme in leading strand synthesis and on a lagging strand model DNA. The results indicate that helicase unwinding limits the rate of the *E. coli* replisome, similar to the case of the bacteriophage systems. *In vivo* cell imaging techniques of live *E. coli* also indicate that the lagging strand is faster than the replisome (24). The cellular studies imaged fluorescent SSB during replication and observed bursts of SSB association/dissociation with DNA. This result suggests the leading strand is slow compared to the lagging strand because if the rate of lagging strand synthesis were equal to the leading stand, occupancy of SSB on the DNA should have remained constant. A replisome that is limited by the rate of helicase unwinding, rather than polymerase extension, ensures that synthesis of lagging strand Okazaki fragments will always be sufficiently fast to keep pace with the leading strand polymerase.

## SUPPLEMENTARY DATA

Supplementary Data are available at NAR Online.

SUPPLEMENTARY DATA
