# Supplementary material for: Replisome mechanics: lagging strand events that influence speed and processivity
Source: Nucleic Acids Res. 2014 May 16;42(10):6497–510. doi: 10.1093/nar/gku257 (PMC4041431; doi:10.1093/nar/gku257)
Supplement: SUPPLEMENTARY DATA [file supp_42_10_6497__index.html]

Replisome mechanics: lagging strand events that influence speed and processivity — SUPPLEMENTARY DATA 

# Replisome mechanics: lagging strand events that influence speed and processivity

## SUPPLEMENTARY DATA

**Files in this Data Supplement:**

- SUPPLEMENTARY DATA
- SUPPLEMENTARY DATA
