# Supplementary material for: Replisome mechanics: lagging strand events that influence speed and processivity
Source: Nucleic Acids Res. 2014 May 16;42(10):6497–510. doi: 10.1093/nar/gku257 (PMC4041431; doi:10.1093/nar/gku257)
Supplement: SUPPLEMENTARY DATA [file supp_gku257_nar-00189-f-2014-File009.docx]

**SUPPLEMENTARY MATERIAL**

**For the manuscript**

**Replisome Mechanics: Lagging Strand Events that Influence Speed and Processivity**

Roxana E. Georgescu^1^, Nina Yao^1^, Chiara Indiani^2^, Olga Yurieva^1^ and Mike E. O’Donnell^1,^*

^1^Rockefeller University

Howard Hughes Medical Institute

1230 York Avenue

New York, NY 10065

^2^ Manhattan College

4513 Manhattan College Pkwy

Riverdale, NY 10471

This supplementary material contains:
6 Supplementary Figures

Description of Supplementary Video 1

**
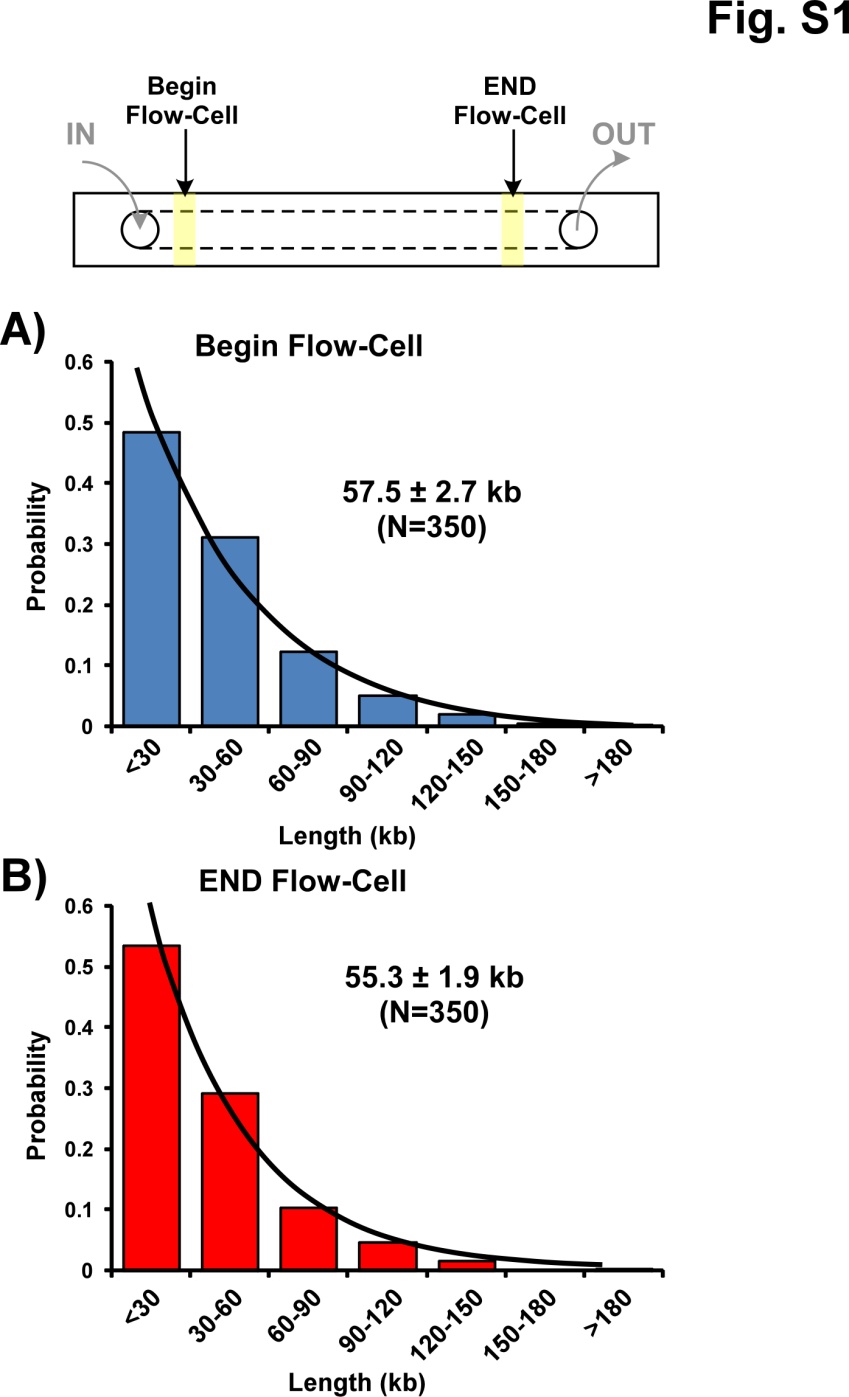
**

**Figure S1. *E. coli* Pol III does not rebind to another DNA substrate once it dissociates.** Panels A and B are DNA length distribution histograms of products analyzed using several visual fields located at the beginning of the flow-cell (A), or at the end of the flow-cell (B) as shown in the scheme above. Numbers represent the single-exponential fit ± S.E.M of the total number (N) of molecules analyzed.


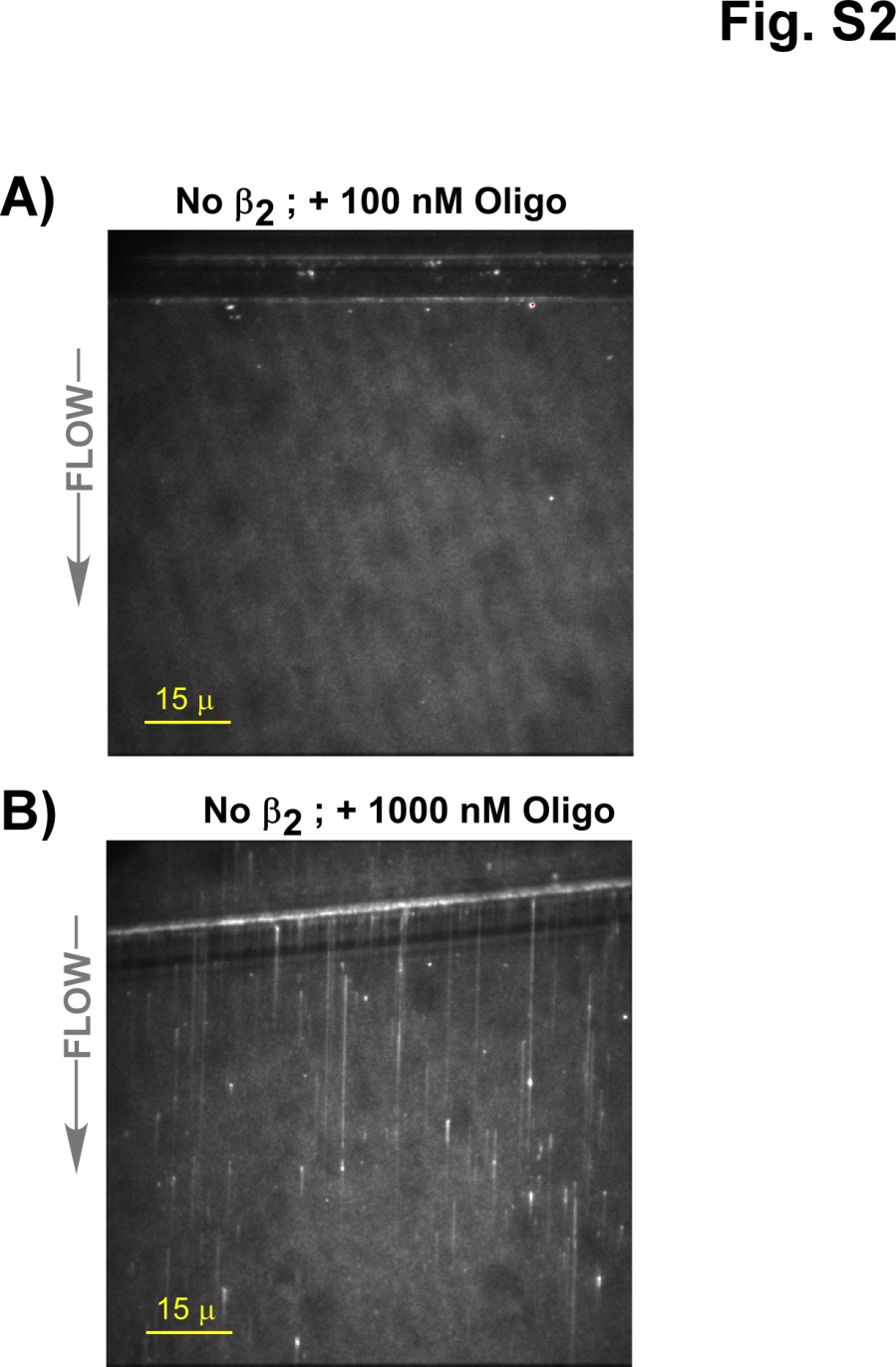


**Figure S2. Control reactions that use a DNA 20mer to prime the lagging strand**. Single-molecule assays utilized a DNA 20mer in place of primase to prime Okazaki fragment synthesis. Panel A: In the absence of β and primase, no DNA product is observed using 100 nM DNA 20mers in the presence of 500 nM SSB_4_. Hence, annealing of the 20mer to DNA is lowered by SSB to a level that cannot be visualized by the fluorescent DNA intercalator, yet anneals with sufficient frequency to prime lagging strand synthesis. Panel B: the concentration of DNA 20mer is increased to 1 μM, a concentration that allows sufficient annealing to DNA for observation by the fluorescent intercalator, thereby showing that the leading strand ssDNA is indeed formed.

**
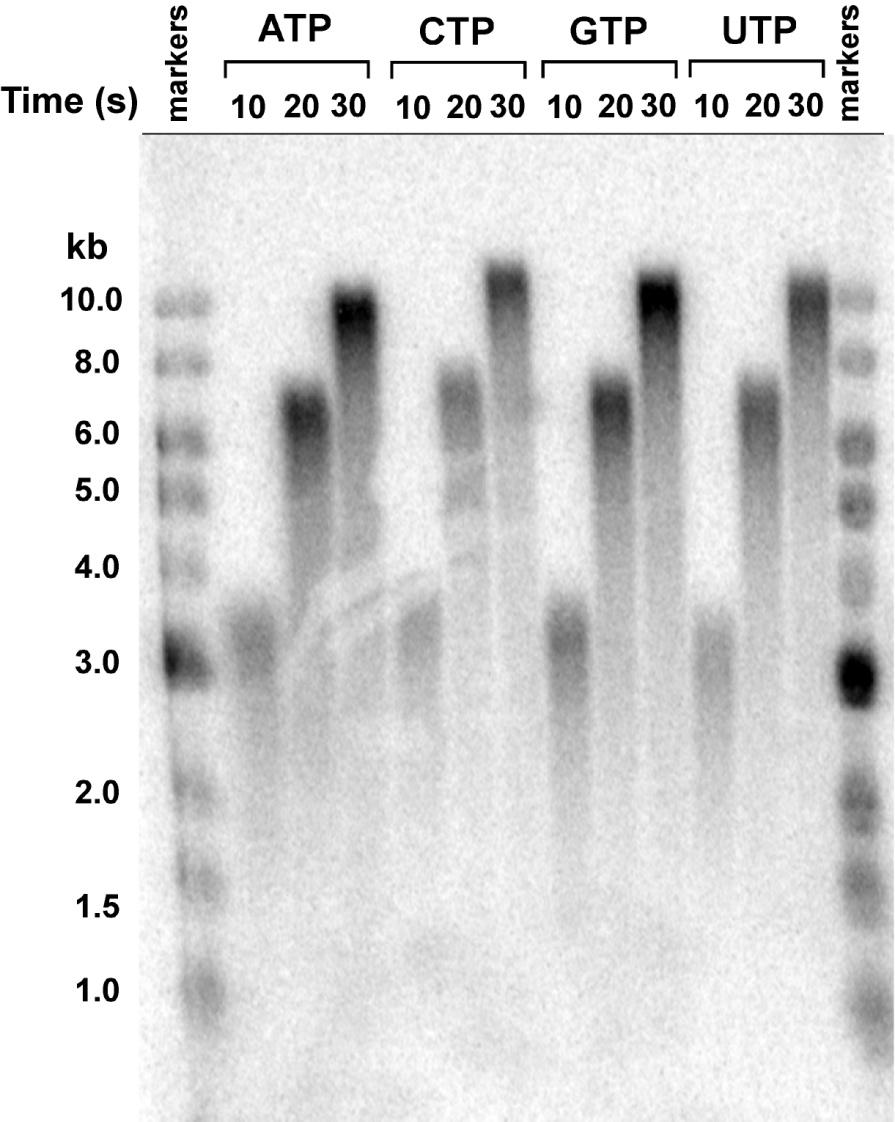
**

**Figure S3. Effect of rNTPs on the rate of replisome.** Coupled leading/lagging strand 100mer rolling circle replication reactions were performed as described in Materials and Methods section. Each of the 4 rNTPs (0.5 mM) is equally capable of supporting DnaB unwinding, and thus rNTPs do not act on the helicase to slow the fork. First and last lanes are size markers.

**
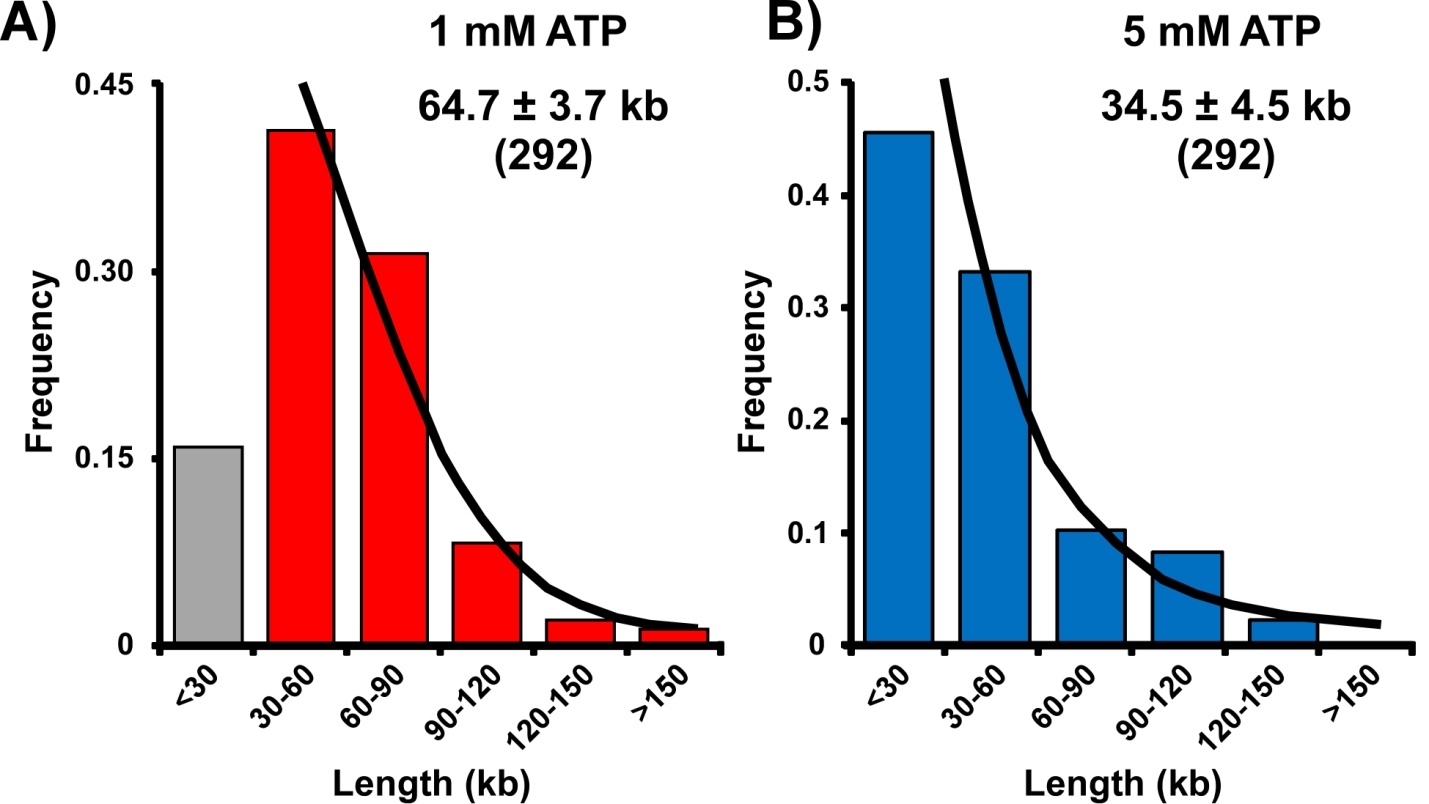
**

**Figure S4. ATP effect on replisome processivity.** Histograms depicting the DNA length distribution obtained from single molecule replication reactions performed at 1 mM ATP (panel A) and 5 mM ATP (panel B). Primase was not present in these assays and therefore the observed decrease in replisome progression is not due to primer synthesis. Numbers represent the single-exponential fit ± S.E.M of the total number (N) of molecules analyzed.

**
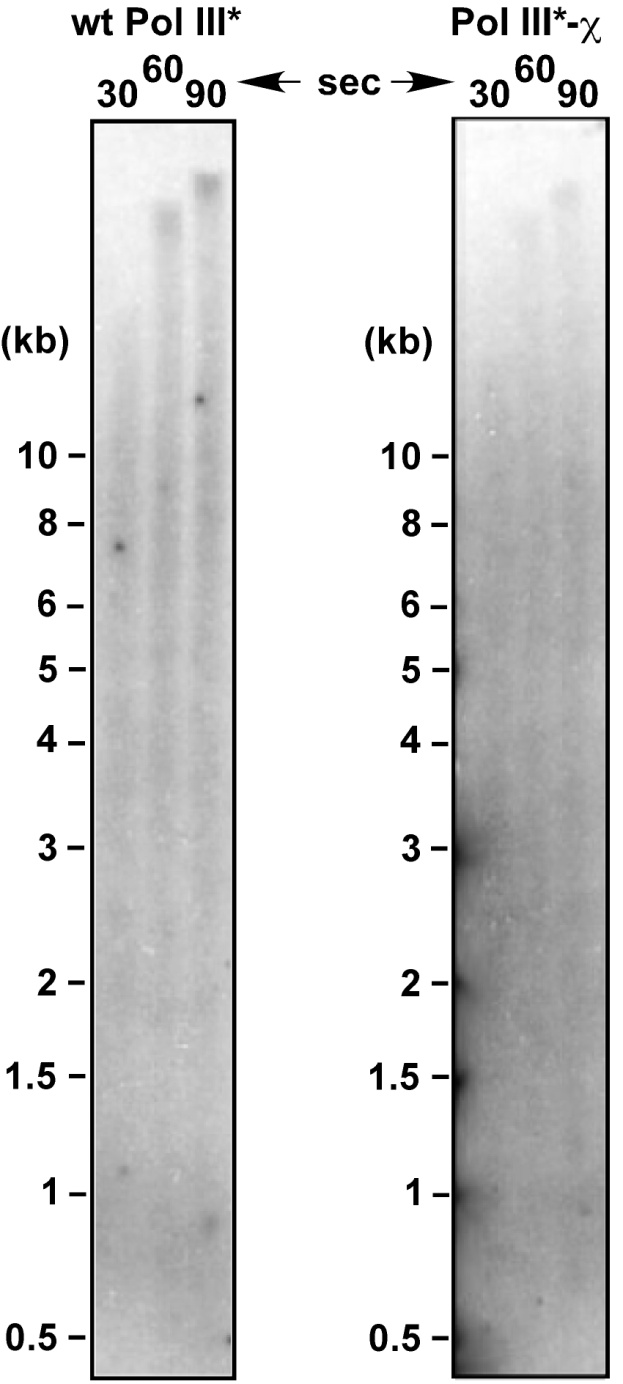
**

**Figure S5. χ-to-SSB interaction enhances replication.** Leading/lagging replication reactions were performed side-by-side using DnaB, β, primase, SSB and either Pol * or Pol III* minus chi. All lanes shown were from the same gel.

**
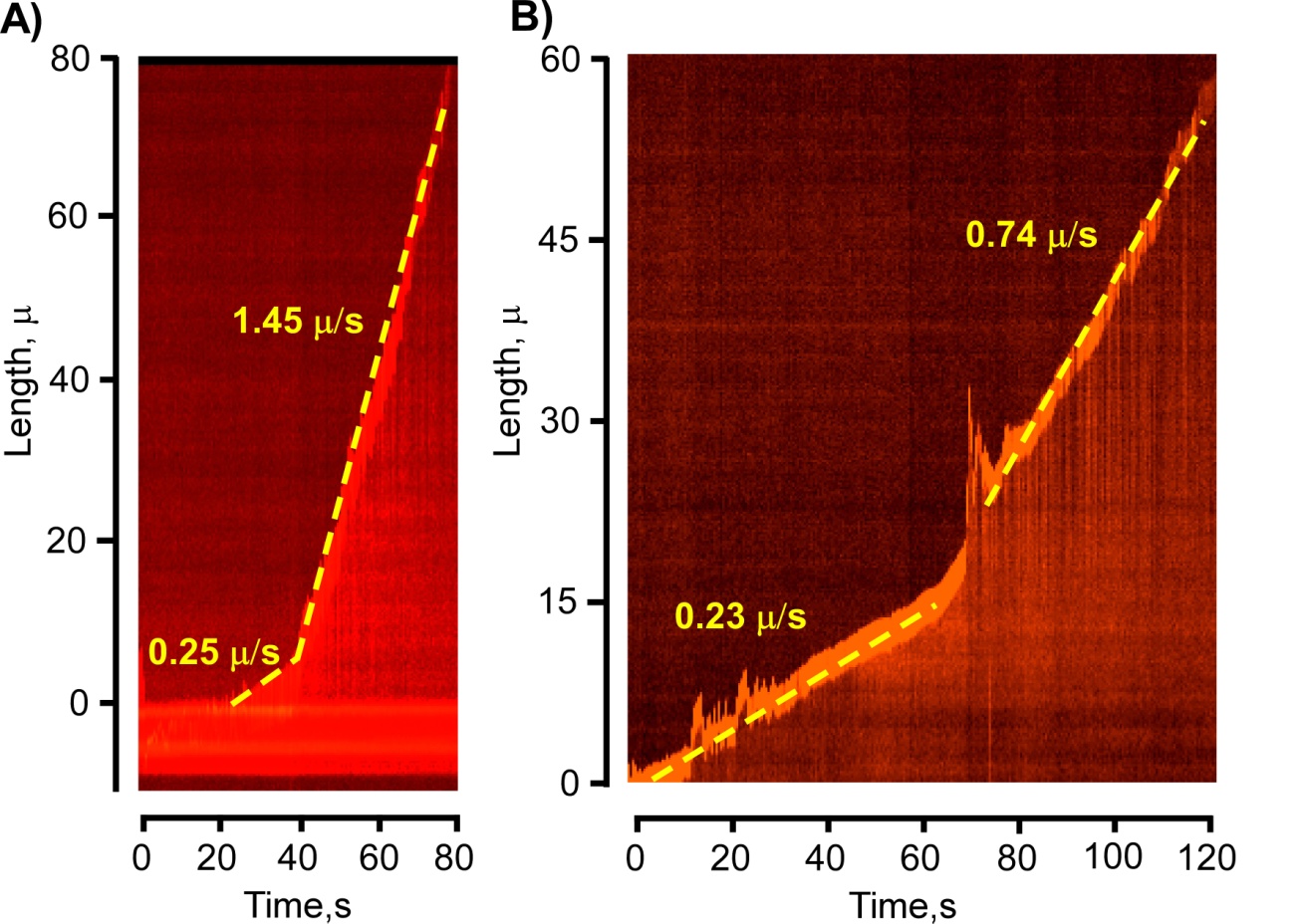
**

**Figure S6. Example kymographs of SSB-ssDNA leading strand products.** Single-molecule 100mer rolling circle replication reactions using Texas red-labeled SSB (continuously present in the flow) are performed under epifluorescence detection conditions. Product length is measured in microns, since the length of ssDNA present in the SSB-ssDNA complex is uncertain. Assuming the same length of ssDNA-SSB as dsDNA, the 1.45 micron/s rate would translate to about 4.5 kb/s. Panels A and B are kymographs of two different molecules and are shown to demonstrate the various rates of rapid burst growth that can occur during the replication reaction.

**Supplementary Movie 1.**

**Use of fluorescent SSB to identify ssDNA in DNA products**. The video depicts one close-up recording of a DNA product of MonoPol replisomes, in which reaction contained fluorescently labeled SSB described in the Materials Methods Section. The video shows that DNA strand contain fluorescently labeled *E. coli* SSB (with Oregon Green488 Maleimide). The duplex DNA is visualized because fluorescent SSB bound to DNA. The movie contains circa 3’ 30” of experimental data rendered at 20 frames per second (original data acquisition is 1 frame/s at 100 ms exposure for each frame).
